# Evaluating Youth Participatory Action Research in the Americas: Comparative Insights on Empowerment, Methodologies, and Social Change

**DOI:** 10.1002/jad.70091

**Published:** 2026-01-10

**Authors:** John Diaz, Paola Velasquez Quintero, Cody Gusto, Ivan Muñoz‐Echeverri, Camilo Norena Herrera, Laura Valencia

**Affiliations:** ^1^ Gulf Coast Research and Education Center University of Florida Plant City Florida USA; ^2^ Department of Agricultural Education and Communication University of Florida Gainesville Florida USA; ^3^ National Faculty of Public Health University of Antioquia Antioquia Colombia; ^4^ Institute of Food and Agricultural Sciences University of Florida Bunnell Florida USA

**Keywords:** adolescent civic engagement, community‐based research, comparative analysis, social justice, youth empowerment, Youth Participatory Action Research (YPAR)

## Abstract

**Introduction:**

Youth Participatory Action Research (YPAR) positions adolescents as co‐researchers to investigate and address social issues affecting their lives. While YPAR has gained global prominence, comparative research examining how it is conceptualized and practiced across regional contexts remains limited. This study analyzes the differences and commonalities in participatory research projects involving youth conducted in North America and Latin America, focusing on theory, methodology, youth participation roles, and socio‐political contexts.

**Methods:**

We conducted a rapid review of 85 peer‐reviewed and grey literature sources published between 2003 and 2023, utilizing the SPIDER framework. Sources were screened through a multi‐step collaborative process and analyzed thematically across four domains: theoretical foundations, methodological design, domains of inquiry, and youth roles, assessed using Arnstein's Ladder of Citizen Participation.

**Results:**

Studies in North America often emphasized individual youth empowerment and institutional reform, drawing on critical pedagogy and positive youth development frameworks within school and health settings. In contrast, Latin America studies prioritized collective action, sociopolitical transformation, and decolonial theory, frequently embedded in grassroots movements. Regarding participation, youth in North America often assumed protagonist roles within structured institutional boundaries, while Latin America projects engaged youth as co‐creators of knowledge within broader community‐driven struggles.

**Conclusions:**

This review highlights significant regional distinctions in how YPAR is theorized, practiced, and institutionalized. Findings suggest that hybrid approaches—integrating the institutional access common in North America with the grassroots, collective activism of Latin America—may enhance the transformative potential of YPAR. Future scholarship should explore context‐sensitive frameworks that elevate youth leadership while respecting regional epistemologies and political realities.

## 1 Introduction

Youth Participatory Action Research (YPAR) is a transformative methodology that centers young people as co‐researchers in processes aimed at social change. Rooted in traditions of critical pedagogy and participatory action research (PAR), YPAR challenges conventional, adult‐centered research paradigms by affirming youth as capable agents of inquiry and action (Cammarota 2017). It draws on foundational scholarship from Paulo Freire (1980), who emphasized education as a practice of freedom, and builds on feminist, decolonial, and critical race theories that highlight the intersectional experiences of marginalized youth (Anyon et al. 2018; Cammarota and Fine 2008).

Over the past two decades, Youth Participatory Action Research (YPAR) has emerged as an increasingly prominent approach across a range of global contexts. In North America, including the United States and Canada, YPAR has been widely incorporated into educational settings and youth development programs as a strategy to advance equity, foster civic engagement, and support identity development among young people (Ozer et al. 2010). In contrast, across Latin America, encompassing Mexico, Central and South America, and the Caribbean, YPAR as a distinct framework is less commonly referenced. Instead, participatory research with young people in these contexts is more often rooted in community‐based movements and draws on traditions of popular education, Latin American critical theory, and decolonial thought (Arce and Fritz 2019; Di Iorio et al. 2021; Muñoz‐Echeverri et al. 2024; Shabel 2014; Voltarelli 2018).

Despite shared commitments to youth empowerment and participatory practice, there are important epistemological, methodological, and political differences in how YPAR is theorized and implemented across the Americas. In the North American context (USA and Canada), YPAR research is often situated within developmental or educational frameworks shaped by neoliberal reforms that emphasize accountability and standardized testing, prioritizing individual responsibility over collective processes (De Lissovoy 2013; Giroux 2014). Structural racism has also produced persistent racialized disparities in access to resources and disciplinary practices (Anyon 1981). These conditions have placed YPAR mainly within institutional frameworks, framing research with young people in contexts of time constraints given the emphasis on school efficiency, as well as the risk of co‐optation, racism, and undervaluation of their knowledge, given that greater credibility is given to “official” knowledge over local knowledge and restrictions on transformations to incremental reforms (Akom et al. 2016; Davis 2016; Desai 2019; Ozer et al. 2013). In contrast, participatory research traditions in Latin America (Central and South America, Mexico, and the Caribbean) have been shaped by histories of colonialism, authoritarian regimes, and social movements resisting neoliberal reforms. As a result, YPAR‐related experiences in the region tend to emphasize collective action, cultural resistance, and community sovereignty, drawing on Freirean popular education, decolonial frameworks, and critical social psychology (Fals‐Borda 1987; Freire 1980; Martín‐Baró 2006; Mota Neto 2018). These distinctions matter because they shape not only the processes of youth engagement but also the types of knowledge produced, and the kind of transformative process pursued.

Yet, to date, there has been no systematic effort to compare YPAR in North America with the distinct traditions of participatory research involving youth utilized in Latin America. Existing literature predominantly centers on national case studies or localized interventions, often within singular cultural or institutional contexts (Anyon et al. 2018; Ozer et al. 2020). While these studies have provided valuable insights into youth engagement and empowerment, they rarely interrogate how regional histories, political ideologies, and academic traditions influence the conceptual and practical contours of YPAR and participatory research with youth. Scholars have highlighted the need for more critical, cross‐regional comparisons that attend to the epistemological and structural dimensions shaping participatory research with youth (Cahill 2007; Cammarota and Fine 2008). This absence of comparative analysis limits the field's ability to understand the contextual factors that support or hinder meaningful youth participation and restricts opportunities for cross‐regional learning and methodological innovation.

The positioning of young people in research and public life is also shaped by international frameworks, particularly the United Nations Convention on the Rights of the Child (CRC). The CRC asserts the right of youth to express their views freely in all matters affecting them and to participate meaningfully in decision‐making processes (United Nations 1989). While all countries in Latin America have ratified the CRC and many have incorporated its principles into national legislation and educational policy, the United States remains the only UN member state that has not ratified the convention (United Nations Treaty Collection n.d.). This discrepancy highlights divergent structural commitments to youth agency and may contribute to regional differences in how YPAR is institutionalized and practiced across the Americas.

To address this gap, this literature review synthesizes peer‐reviewed YPAR studies conducted in North America (Canada and the United States) alongside participatory research with young people carried out in Latin America (Mexico, Central and South America, and the Caribbean). While Mexico is geographically part of North America, we included it in the Latin American sample due to its strong cultural, linguistic, and historical affinities with Central and South American countries (Knight 2002; Saldaña‐Portillo 2003). This decision aligns with longstanding conventions in Latin American studies, which often conceptualize Mexico as part of Latin America based on shared colonial histories, language, and sociopolitical trajectories (Mignolo 2005; Raja et al. 2013). It also reflects the regionally grounded pedagogical traditions that inform participatory research in Mexico, including popular education, liberation pedagogy, and community‐based youth engagement (Briseño Roa 2018). By examining the similarities and differences in the theoretical and political orientations of the research, the ways in which youth and their participation are conceptualized, the methodological designs and modes of youth engagement, and the purposes of the studies, we seek to offer new perspectives on the global diversity of YPAR and participatory research with young people. Our intention is not to impose a singular definition or hierarchy of YPAR approaches, but rather to illuminate the varied ways youth participate in knowledge production and social change across distinct socio‐political landscapes.

This absence of comparative analysis limits the field's ability to understand the contextual factors that support or hinder meaningful youth participation and restricts opportunities for cross‐regional learning. This review was undertaken by a binational team of researchers committed to participatory approaches in youth development across the Americas. The study emerged from a U.S.–Colombia research partnership focused on promoting transnational collaboration and deepening understanding of how regional socio‐political and epistemological contexts shape youth participatory research. Drawing from the team's lived and academic experiences in both Latin America and North America, this review not only aims to describe the differences in the implementation across the Americas but also to critically reflect on what these variations reveal about methodological traditions and the diverse conceptions of youth agency in the region.

This review is guided by four research questions

1. How are youth and their participation conceptualized in YPAR and in participatory research with young people in Latin America?
2. What are the similarities and differences between YPAR in North America and participatory research with young people in Latin America with respect to the theoretical and political orientations of the studies?
3. What are the methodological similarities and differences—particularly regarding the involvement of youth in the research process—between YPAR in North America and participatory research with young people in Latin America?

Do YPAR in North America and participatory research with young people in Latin America differ in terms of their purposes and praxis?In pursuing these questions, this article contributes to the growing field of youth‐led research by offering a comparative lens that respects both regional specificity and global interconnectedness. It also responds to calls from scholars and practitioners to more rigorously examine the assumptions and practices underlying participatory work with youth (Cahill 2007; Ozer et al. 2020). The findings are intended to inform future YPAR practice, support context‐sensitive adaptations, and foster transnational dialogue among researchers, educators, and youth collaborators.

## 2 Methods

### 2.1 Research Design

A rapid review of peer‐reviewed and grey literature, including meta‐analyses and systematic reviews, focused on YPAR, PAR, and Community‐Based Participatory Research (CBPR) projects involving youth in the Americas. A rapid review, also known as a Quick Review of Evidence, is a form of evidence synthesis that provides a timely summary of existing research by streamlining search criteria or limiting the number of databases queried (Langlois et al. 2019; Tricco et al. 2022). This approach enables efficient evidence synthesis to support decision‐making while upholding methodological rigor and transparency (Garritty et al. 2021).

We focused on studies based in North America and Latin America that addressed participatory research involving young people within various domains, including youth health equity, juvenile justice reform, youth development, and educational empowerment, intending to synthesize relevant findings and develop recommendations. This study is a literature review that synthesizes information from previously published sources and does not involve human participants, animal subjects, or primary data collection. Therefore, ethical approval was not required.

### 2.2 Literature Search Strategy

We applied the SPIDER framework (Sample, Phenomenon of Interest, Design, Evaluation, Research type) to develop a comprehensive search strategy for identifying relevant YPAR literature. Between August and October 2024, we conducted systematic searches across multiple academic databases, including APA PsycInfo, Web of Science, Google Scholar, ProQuest Dissertations & Theses Global, BIREME LILACS, Dialnet, La Referencia, and institutional repositories such as the Universidad de Antioquia Digital Library. Searches were limited to the years 2003–2023 and to studies focused on the Americas, with inclusion criteria requiring publications in English, Spanish, or Portuguese.

The search strategy used combinations of terms related to youth populations, participatory research approaches, and relevant theoretical orientations. For North American sources, terms included: *Youth Participatory Action Research*, *Community‐Based Participatory Research*, *CBPR*, *youth organizing*, *youth‐led*, *critical pedagogy*, *citizen science*, *youth participation*, *youth empowerment*, *adolescent health*, *youth development*, *youth civic engagement*, and *school‐based participatory research*. These were paired with demographic descriptors such as *adolescents*, *youth*, *young people*, *teenagers*, and *young adults*. Boolean search string combinations such as (“youth‐led” OR “adolescents” OR “young people”) AND (“YPAR” OR “CBPR” OR “critical pedagogy”) were used to identify relevant studies across platforms.

For Latin American sources, equivalent terms were applied in Spanish and Portuguese to ensure cultural and linguistic relevance. These included: *Investigación Participativa Basada en la Comunidad*, *Investigación Acción Participativa con jóvenes*, *Investigación Participativa*, *participación juvenil*, *liderazgo juvenil*, *empoderamiento*, *Desarrollo humano*, *Ciencia Ciudadana*, and population terms such as *Adolescente*, *Jóvenes*, *Niño*, *Menores*, and *Adulto Joven*. For example, compound search expressions included combinations like: (Adolescente OR Jóvenes) AND “Investigación Acción Participativa” AND (empoderamiento OR liderazgo), and (“Adulto joven” OR Adolescente) AND “Investigación Participativa Basada en la Comunidad.”

Importantly, the term *Youth Participatory Action Research* (and its acronym, *YPAR*) is not widely used in Latin American scholarship despite the presence of parallel practices rooted in popular education and participatory traditions. As noted by Tandon et al. (2016), terminology and frameworks developed in the Global North may not align with the lexicon or conceptual framing used in Latin American community‐based research. Consequently, we broadened our search strategy to include culturally and linguistically resonant terms that reflect the region's theoretical grounding in *investigación acción participativa* and *educación popular*, ensuring that the review captured substantive youth‐led participatory work regardless of labeling conventions.

### 2.3 Inclusion and Exclusion Criteria

To be included in this review, articles were required to meet a set of methodological, geographic, and linguistic criteria. First, studies had to be published in a peer‐reviewed journal between 2000 and 2024 to ensure contemporary relevance and consistency with the evolution of YPAR practices. Eligible studies needed to report on research projects in which youth between the ages of 10 and 24 were engaged as co‐researchers or collaborators, rather than solely serving as participants or data sources. Inclusion was limited to studies that explicitly aligned with the core principles of PAR—specifically those emphasizing co‐construction of knowledge, critical reflection, youth‐led inquiry, and a commitment to social action or community change.

Geographic scope was restricted to studies conducted in North America (including the United States and Canada) and Latin America, to allow for a regional comparison of YPAR approaches. For the purposes of this review, Latin America includes Mexico, Central America, South America, and the Caribbean. Only articles that provided sufficient methodological detail to assess the nature and depth of youth involvement, the study's theoretical orientation, and the participatory structures used were considered. Finally, to ensure accessibility and accurate interpretation, all articles had to be available in English, Spanish, or Portuguese.

Studies were excluded if they focused on youth only as research subjects, described theoretical or conceptual models without empirical data, or addressed participatory evaluation or design processes without actual youth collaboration in research activities.

### 2.4 Screening and Selection Process

To ensure that only high‐quality studies were included in the review, we conducted a rigorous quality appraisal using a criteria checklist adapted from the Joanna Briggs Institute (JBI) Manual for Evidence Synthesis (Aromataris et al. 2024). This checklist was tailored for our review of participatory qualitative research and included multiple indicators drawn from JBI's Critical Appraisal Checklist for Qualitative Research, such as the congruity between stated philosophical perspective and methodology; the alignment between methodology and research questions, methods, data representation, and interpretation; the articulation of researcher influence and reflexivity; and the presence of ethical considerations and approval. Additional checklist items considered for our purposes included the clarity of youth roles in the research process and evidence of co‐construction or critical reflection—essential markers for credible YPAR work.

The initial database search yielded 412 articles. After duplicates were removed and an initial screening was conducted, 206 articles were retained for full‐text quality assessment. Each full‐text article was independently assessed by two reviewers to determine whether it met the threshold for methodological transparency, rigor, and participatory integrity. Studies that failed to provide adequate information on participatory structures or demonstrated only superficial youth involvement (e.g., youth as data collectors only) were excluded. Importantly, methodological diversity was preserved by including studies with a range of qualitative, mixed‐methods, and action‐oriented approaches, as long as they met the adapted JBI standards for quality and participation.

After completing the quality assessment, the research team systematically identified and selected relevant resources. They managed eligible sources using Zotero referencing software (Zotero, version 7) and documented them in a Microsoft Excel spreadsheet for organization and categorization. Within the Excel review matrix, resources were initially ranked based on type, publication date, methodology, target audience, and other relevant criteria. Screening and selection were conducted through a multi‐phase, team‐based process grounded in peer debriefing and analyst triangulation. Two lead reviewers, one for North America and one for Latin America, conducted independent initial screenings and prepared structured debriefing documents summarizing eligible studies. These documents were presented to the full research team for group feedback. Inclusion decisions were based on full team consensus; if agreement could not be reached following discussion, the article was excluded. Multiple reviewers were involved at each stage of selection and analysis, which enhanced the transparency and credibility of decisions through analyst triangulation (Carter et al. 2014). Discrepancies in coding or inclusion were resolved through deliberation, and only studies meeting the adapted inclusion criteria and achieving consensus were retained for full review.

A total of 85 articles were approved for inclusion through team consensus, following full‐text review and quality appraisal. Of these, 56 articles originated from North America (Canada and the United States), and 29 articles were from Latin America (including Mexico, Central and South America, and the Caribbean).

### 2.5 Coding and Data Extraction

To guide consistent data extraction and support comparative analysis, the research team developed and used a structured worksheet that served as both a coding protocol and evidence matrix. The worksheet included key bibliographic and contextual fields (e.g., authors, institutions, publication venue, year, database source) as well as analytical categories aligned with our review objectives. These included article type, geographic focus, theoretical orientation, participatory structures, youth roles, and stated outcomes. Additional fields captured author‐defined keywords, stated research objectives, and methodological notes.

This worksheet matrix tool enabled the team to systematically compare studies across regions and identify patterns. The worksheet also served as a shared reference document across researchers, supporting reliability in coding and synthesis. Data entered into the worksheet were used to inform thematic analysis.

To ensure analytic rigor and collective interpretation of findings, we employed a peer debriefing process throughout data extraction and thematic synthesis. Coding responsibilities were divided regionally, with one researcher assigned to lead coding for Latin American studies and another for North American studies. This division allowed coders to bring regional, linguistic, and contextual expertise to their initial analyses. After completing preliminary coding, each regional coder developed analytic memos summarizing emerging patterns, themes, and interpretive questions.

These memos were shared with the full author team in peer debriefing sessions designed to interrogate assumptions, validate theme definitions, and refine emergent categories. During these sessions, the team reviewed the coded data collaboratively and offered critical feedback on interpretation, gaps, and coding consistency. This iterative process helped surface nuances in how YPAR and participatory research with young people was conceptualized and implemented across regional contexts.

Final decisions regarding the naming, inclusion, or revision of themes were made through consensus. A three‐quarters (75%) agreement rule was used to confirm theme validity or resolve discrepancies among authors. This participatory and team‐based approach to analysis ensured that findings reflected collective insights and mitigated the influence of individual coder bias. It also allowed for greater reflexivity and alignment with the values of participatory inquiry that underpin the YPAR framework itself.

A score of youth participation was also calculated for each article using Arnstein (1969) “Ladder of Citizen Participation” as the theoretical guiding evaluative framework. This approach involved assigning each study a score based on the degree of youth involvement across critical research stages, including planning, design, analysis, dissemination, and transformation. These scores enabled a clear classification of youth participation levels, ranging from “Protagonism,” representing full youth leadership and control, to “Informants,” reflecting minimal involvement and influence in the research process. The research team incorporated these results into the established group debriefing process, revisiting initial coding decisions and using a consensus‐based approach outlined earlier in the study to ensure consistency and accuracy in classifying youth participation.

In assessing youth roles and the degree of participation, we also considered how studies conceptualized youth empowerment and youth leadership. Drawing on Zimmerman (2000) theory of psychological empowerment and Freirean concepts of critical consciousness and dialogical learning (Freire 1980), we interpreted empowerment as a combination of intrapersonal, interactional, and behavioral elements—such as increased self‐efficacy, decision‐making agency, and engagement in social action. Youth leadership was defined as active involvement in shaping the research process, including planning, design, data interpretation, and the transformative actions. These constructs were integrated into our coding framework to help differentiate between projects that positioned youth as participants versus co‐researchers or movement leaders.

### 2.6 Analytical Strategy

Once coding was completed, the research team engaged in a structured cross‐case thematic analysis to identify patterns of convergence and divergence in how YPAR is conceptualized and implemented across NA and LA. The analysis was guided by four overarching domains derived from the literature and our research questions:

1. Conceptualization of youth and participation
2. Theoretical and political orientation
3. Methodological structures and youth involvement
4. Purpose and praxis of YPAR or participatory research with young people

To deepen the credibility and trustworthiness of the findings, the cross‐case analysis was subjected to a second round of peer debriefing. During this phase, draft thematic summaries for each domain were presented to the broader author team, who reviewed the patterns emerging within and across regions. Authors provided critical feedback on theme definitions, boundaries, and salience, with particular attention to whether the synthesis accurately captured the distinct contextual, cultural, and ideological dimensions of YPAR in each region.

Consensus was used as the guiding principle for analytic decisions. Themes or comparisons were only retained if at least three‐quarters of the author team agreed on their validity and clarity. This iterative, participatory process allowed for reflexive engagement and minimized individual interpretive bias.

Following the peer debriefing and revision process, a final whole‐group read‐through of the written results was conducted. Authors reviewed the drafted narrative as a collective, and identified areas needing clarification, additional citations, or conceptual refinement. Final revisions were made based on consensus, ensuring that the analytical narrative reflected both the evidence from the reviewed studies and the collective interpretation of the research team.

### 2.7 Positionality Statement

This study was led by two researchers with extensive experience in YPAR and participatory methodologies. The first principal investigator has worked with Latino communities and focuses on adapting and applying best practices from LA participatory approaches to serve LA audiences in the United States. This work seeks to bridge cultural and geographical gaps in educational practices and policies while fostering transnational learning and collaboration.

The second principal investigator leads a large PAR initiative focused on intergenerational collaboration in research, with an emphasis on critical pedagogy, popular education, and decolonial methodologies. This initiative promotes youth involvement in research and has created opportunities for intergenerational dialogue, supporting participatory methodologies and community‐based knowledge production.

The two principal investigators were brought together by collaborative projects funded by the Department of State, which aimed to strengthen academic and research connections between the United States and Colombia. This collaboration has fostered opportunities to better understand how participatory approaches can be improved, how they differ across regions, and how they can be integrated for better transnational collaboration.

The researchers acknowledge that their positionalities—as practitioners working in both North and LA contexts—shape their perspectives and interpretations. This collaboration is guided by a shared commitment to elevating youth voice, honoring epistemological diversity, and fostering cross‐regional dialogue in the field of YPAR. Graduate student co‐authors, who represent multiple national and disciplinary backgrounds, contributed to the coding, analysis, and interpretation, enriching the analytic process and supporting the study's commitment to reflexivity and methodological transparency.

## 3 Results

This section presents the findings from the analysis of 85 participatory research studies involving young people conducted in NA and LA (see Table 1). The results are organized around four research questions and key thematic domains: (1) conceptualization of youth and youth participation, (2) theoretical and political orientation, (3) methodological structures and youth involvement, and (4) purpose and praxis of YPAR. Each domain integrates relevant citations from across the studies to reflect the comprehensive insights drawn from this body of literature.

**Table 1:** Final list of articles from LA and NA included in the analysis including country of focus, theoretical framework, methodology and main topic/issue.

Region	Country	Citation	Theoretical framework	Methodology	Main topic/issue
LA	Uruguay	Ariza‐Arias (2018)	Popular Education, Human Needs theory	Participatory case study (youth cooperative)	Rural youth land access and agroecology
LA	Colombia	Barrantes‐Rojas (2012)	Constructionist learning theory	Case study (educational intervention with video game)	Technology and cognitive skills in education
LA	Brazil	Brandão Neto (2018)	Freirean critical pedagogy	Action research (anti‐bullying program design)	School bullying prevention (culture circles)
LA	Brazil	Brandão Neto et al. (2020)	Critical pedagogy, Youth empowerment	Program evaluation (intervention study)	Bullying prevention with youth leaders
LA	Brazil	Costa et al. (2013)	Health promotion, Social representation	Participatory qualitative study	Rural adolescents' perceptions of health
LA	Colombia	Esguerra‐Moreno and Roa‐Velandia (2015)	Critical pedagogy (affective focus)	Participatory design research (school‐based)	Enhancing child participation in school (affective approach)
LA	Brazil	Frutuoso et al. (2022)	Rights‐based (right to food), Empowerment	Community‐based participatory project	Child nutrition and collective action (SDG goals)
LA	Colombia	Gómez‐Ramírez et al. (2023)	Popular Education, Narrative inquiry	Participatory case analysis	Youth as community researchers in an urban periphery
LA	Costa Rica	Gómez‐Villota et al. (2015)	Community participation theory	Community‐Based Participatory Research	Youth sexual and reproductive health
LA	Bolivia	González‐Morales (2019)	Transdisciplinarity, Decolonial theory	Participatory action research	Youth and food sovereignty in rural communities
LA	Colombia	González‐Quiroz and Pérez‐Cadavid (2018)	Popular Education, Political participation	Community case study (systematization)	Urban youth political organizing (Medellín)
LA	Colombia	Hincapié and Herrera (2014)	Social inclusion theory	Comparative case study (formal vs. informal education)	Play and physical activities for youth civic engagement
LA	Brazil	Iuri et al. (2019)	Popular education (assumed)	Community‐based participatory project	Youth empowerment and advocacy (Brazilian context)
LA	Mexico	JiménezVallejo and ValadezFigueroa (2003)	Community health education	Participatory diagnosis (surveys & workshops)	Adolescent alcohol use in a secondary school
LA	Colombia	Lince‐Bohórquez (2010)	Critical pedagogy	Qualitative case study	Critical consciousness in former student leaders
LA	Colombia	López‐Taborda (2019)	Popular Education	Systematization of practice	Urban youth community project (Nuevo Occidente, Medellín)
LA	Colombia	Luján‐Cortés (2020)	Popular Education, Cultural studies	Narrative case study	Youth transformation through community arts
LA	Colombia	Naranjo‐Rojo et al. (2022)	Historical memory, Freirean pedagogy	Action research (educational intervention)	Collective memory and cultural heritage education
LA	Colombia	Niquepa (2023)	Community participation theory	Action research (youth training program)	Strengthening youth civic engagement (local council)
LA	Colombia	Novoa and y Poveda Garrido (2020)	Financial education theory	Case study (classroom project)	Youth financial literacy and savings habits
LA	Colombia	Ospina‐Álvarez (2022)	Arts‐based education, Gender theory	Participatory education project (arts curriculum)	Fostering LGBTQ+ empathy via school arts program
LA	Colombia	Otálvaro Castro et al. (2022)	Knowledge democracy, Public engagement	Case study (knowledge co‐production initiative)	Youth co‐creation of knowledge and policy
LA	Colombia	Pérez‐Velásquez (2019)	Decolonial theory, Indigenous rights	Community case study (qualitative)	Indigenous youth and territorial governance
LA	Colombia	Restrepo Galvis et al. (2023)	Decolonial feminist lens	Qualitative field study	Child‐rearing practices & sexual rights (indigenous community)
LA	Colombia	Rodríguez‐Gaitán (2020)	Participatory research training, Decolonial	Participatory case study (youth training)	Youth research on territorial conflicts
LA	Colombia	Téllez‐Ballén (2019)	Popular Education (hope theory)	Systematization of community experience	Community education and cultures of hope
LA	Colombia	Urrea‐Pérez (2014)	Popular Education, Civic engagement	Systematization of an experience	Community education for citizen participation
LA	Colombia	Vanderbilt Hernández (2020)	Arts in education	Intervention case (school drama program)	Theater‐based approach to bullying
LA	Colombia	Vanegas (2018)	Critical youth citizenship	Qualitative case study	Political subjectivity in NGO youth participants
NA	United States	Akom et al. (2008)	Critical youth studies, Critical pedagogy	Conceptual essay/theory analysis	Critical youth empowerment theory
NA	United States	Akom et al. (2016)	Critical pedagogy, Digital media theory	Case study (food justice campaign)	Food justice and technology
NA	United States	Anderson (2020)	Youth empowerment (implicit)	Systematic review (qualitative)	YPAR in high school settings
NA	United States	Anderson et al. (2024)	Critical consciousness (Freire)	Case study (YPAR project)	Food security and youth activism
NA	United States	Anyon et al. (2018)	Positive youth development	Systematic review	YPAR outcomes in U.S. contexts
NA	Canada	Branquinho et al. (2020)	Community psychology, Youth health promotion	Systematic review	Youth health and well‐being
NA	United States	Brown (2010)	Critical pedagogy, Youth–adult partnership	Action research case study	School discipline reform
NA	United States	Call‐Cummings and Martinez (2016a)	Critical pedagogy, Reflexive practice	Case study (YPAR process critique)	Power dynamics in YPAR
NA	United States	Call‐Cummings and Martinez (2016a)	Critical Race Theory	Case analysis (teacher–student dialogue)	Racial dynamics in education
NA	United States	Cammarota (2008)	Critical ethnography, Freirean praxis	Participatory ethnography	Latinx youth cultural identity
NA	United States	Cammarota (2008)	Social justice education	Action research in curriculum	Equitable curriculum design
NA	United States	Cammarota (2017)	Critical youth studies	Theoretical analysis	Transformational resistance pedagogy
NA	United States	Cammarota and Fine (2008)	Critical pedagogy	Theoretical exposition	YPAR as transformational resistance
NA	United States	Cammarota and Romero (2009)	Latina/o critical theory	Conceptual discussion	Transforming public education
NA	United States	Cammarota and Romero (2011)	Critical pedagogy, Youth empowerment	Case study (school program)	Education policy change
NA	Canada	Conrad (2015)	Cultural democracy, Popular theatre	Case study (arts‐based YPAR)	Arts and social inclusion
NA	United States	Cox (2017)	Youth empowerment theory	Quasi‐experimental (pre–post design)	Student self‐efficacy in school
NA	United States	Davis (2016)	Black feminist thought, STEM education	Participatory case study	Girls' STEM education experiences
NA	United States	deLara (2002)	School safety theory	Survey & focus groups	Peer strategies for school safety
NA	United States	Desai (2019)	Critical PAR pedagogy	Methodological review	YPAR methods and challenges
NA	United States	Desai and Abeita (2017)	TribalCrit, Critical Race Theory	Single‐case narrative	Juvenile justice and self‐advocacy
NA	United States	Fine (2009)	Empowerment theory	Community‐based intervention study	Substance use and HIV prevention
NA	United States	Fisette and Walton (2011)	Feminist empowerment	Case study (photovoice in PE class)	Girls' empowerment in physical education
NA	United States	Foster‐Fishman et al. (2005)	Dialogic participation	Qualitative action research	Youth voice and reflection
NA	United States	Foster‐Fishman et al. (2010)	Youth development, Systems change	Methodological case study	Community change initiatives
NA	United States	Ginwright (2007)	Critical social capital theory	Qualitative case study	Black youth community activism
NA	Canada	Goessling and Doyle (2009)	Arts‐based participatory therapy	Photovoice project	Urban youth mental health
NA	Canada	Goodnough (2014)	Communities of practice (Wenger)	Case study (youth‐led project)	Student‐led educational change
NA	United States	Grace and Langhout (2014)	Critical participatory evaluation	Process evaluation study	Assessing YPAR facilitation
NA	United States	Haskie‐Mendoza et al. (2018)	Healing‐informed, Feminist theory	Case study (program with youth)	Trauma healing with system‐involved youth
NA	United States	Jacquez et al. (2013)	CBPR theory	Literature review (meta‐analysis)	Youth roles in research (various projects)
NA	United States	King (2013)	Critical literacy	Participatory action research (dissertation)	Literacy engagement in high school
NA	United States	Kirshner et al. (2011)	Social cognitive theory (bias awareness)	Case study (school YPAR team)	Student inquiry into bias
NA	United States	Kirshner and Pozzoboni (2011)	Student voice theory	Qualitative case (school closure)	Youth perspective in school reform
NA	United States	Kohfeldt et al. (2011)	Critical youth empowerment	Comparative case study	School‐based YPAR tensions
NA	United States	Levy et al. (2023)	School counseling theory	Scoping review	YPAR in school counseling practice
NA	United States	LPC Consulting Associates, Inc (2015)	— (general YPAR concepts)	Literature review	General YPAR benefits (U.S. context)
NA	United States	Maker Castro et al. (2022)	Critical civic engagement	Multi‐case qualitative study	Immigrant youth civic participation
NA	United States	Malorni et al. (2022)	Positive youth development	Scoping review	Mentoring relationships in YPAR
NA	United States	Nakanishi (2009)	Community psychology	Multi‐case reflection	Facilitation strategies in YPAR
NA	United States	Ozer et al. (2020)	Health equity framework	Integrative review	YPAR for health equity outcomes
NA	United States	Ozer and Douglas (2013)	Empowerment theory	Experimental study (RCT)	Impact of YPAR on youth (civic outcomes)
NA	United States	Ozer et al. (2013)	Institutional theory (power dynamics)	Cross‐case qualitative analysis	Limits of youth leadership in schools
NA	United States	Raygoza (2016)	Critical math pedagogy	Case study (classroom YPAR)	Mathematics education reform
NA	United States	Rubin et al. (2017)	Critical pedagogy	Case study (classroom project)	Implementing YPAR in curriculum
NA	United States	Stovall and Delgado (2009)	Critical legal studies, Freirean praxis	Case study (youth law research program)	Legal literacy and youth rights
NA	United States	S. J. Tanner (2017)	Critical Whiteness pedagogy	Action research (classroom‐based)	Anti‐racist education with white youth
NA	United States	S. Tanner and Corrie (2016)	Critical dialogue (Freire)	Dialogic narrative (teacher–student co‐authorship)	Student–teacher critical dialogue
NA	United States	Vaccarino‐Ruiz et al. (2022)	Decolonial feminist pedagogy	Multi‐method case (photovoice, social biography)	Child‐led inquiry in diverse communities
NA	United States	Valdez et al. (2019)	Prevention science	Systematic review	YPAR in substance abuse prevention
NA	United States	Wang and Pies (2004)	Photovoice (empowerment)	Community‐based photovoice project	Maternal/child health advocacy
NA	United States	Wanis (2010)	Community engagement theory	Multi‐case study (comparative)	Youth influence in public health policy
NA	United States	Wilson et al. (2007)	Empowerment, Photovoice	Participatory action (photovoice)	Youth advocacy on community issues
NA	United States	Wilson et al. (2008)	Dissemination science, PAR	Cross‐site implementation study	Scaling youth‐led research in schools
NA	United States	Yang (2009)	Critical literacy, Ethnomathematics	Case study (class project)	Culturally relevant math education
NA	United States	Zenkov et al. (2013)	Culturally relevant pedagogy	Photographic inquiry (classroom)	Urban literacy practices through photography

### 3.1 Conceptualization of Youth and Youth Participation

Our first research question asked how youth and their participation are conceptualized in YPAR and in participatory research with young people in Latin America. The findings indicate that, in both North America and Latin America, YPAR is grounded in critical traditions that position young people as active contributors to knowledge production and social change. However, the ways in which youth and their roles in YPAR are conceptualized differ in important ways across North American and Latin American contexts. Table 2 presents a comparative analysis of how youth participation in research processes is conceptualized in studies from North America and Latin America.

**Table 2:** Comparison of conceptualizations of youth and their participation in research processes in North American and Latin American studies.

Region	Conceptualization of youth	Representative studies	Illustrative quotes
North America (NA)	Subject of individual agency and voice	Cammarota and Romero (2009), Jacquez et al. (2013), Malorni et al. (2022), King (2013), Rubin et al. (2017)	YPAR facilitates “redefinitions [that] are necessary for young people to feel capable and competent as agents of change” (Cammarota and Romero 2009, p. 64)
	Agent of incremental reform	Cammarota and Romero (2011), Levy et al. (2023), Ozer et al. (2013)	“The present study examines several key questions regarding the potentially ‘bounded’ nature of YPAR in public school settings. First, and most broadly, to what extent are students sufficiently ‘free’ versus ‘bounded’ to engage in YPAR inquiry and change processes …” (Ozer et al. 2013, p. 14)
	Co‐Researcher/Knowledge Creator	Anderson (2020), Anyon et al. (2018), Kirshner and Pozzoboni (2011), Vaccarino‐Ruiz et al. (2022)	“Youth participatory action research (YPAR) involves young people constructing knowledge by identifying, researching, and addressing social problems” (Anyon et al. 2018, p. 865)
Latin America (LA)	Contextual and Dynamic Identity	González‐Morales (2019), Iuri et al. (2019), Lince‐Bohórquez (2010), Vanegas (2018)	“La categoría de joven es sin duda alguna polémica, polifónica, contextual y por ende difícil de enfrentar, enmarcar o definir en parámetros estrictamente delimitados, …, Por lo tanto, para los fines de este trabajo, al hablar de joven se hace necesario hablar de contexto social y contexto histórico” (Vanegas 2018, p. 26) [“The category of youth is undoubtedly controversial, polyphonic, contextual and therefore difficult to confront, frame or define in strictly delimited parameters, …, Therefore, for the purposes of this work, when talking about youth it is necessary to talk about social context and historical context” (Vanegas 2018, p. 26)]
	Collective Sociopolitical Agent	Ariza‐Arias (2018), Niquepa (2023), González‐Quiroz and Pérez‐Cadavid (2018)	“…la historia se puede modificar a través de un proceso educativo que forme una revolución y para ello la juventud es importantísima, por su potencial creador que bien enfocado es propicio para el cambio social” (González‐Quiroz and Pérez‐Cadavid 2018, p. 134) [“…history can be changed through an educational process that forms a revolution, and for this, youth is extremely important, because of its creative potential which, when properly focused, is conducive to social change” (González‐Quiroz and Pérez‐Cadavid 2018, p. 134)]
	Co‐creator of Situated Knowledge	Esguerra‐Moreno and Roa‐Velandia (2015), Gómez‐Ramírez et al. (2023), López‐Taborda (2019), Rodríguez‐Gaitán (2020)	“Este artículo destaca entre los resultados la experiencia de una joven de la comunidad, estudiante universitaria y la emergencia de su habitus como investigadora comunitaria y sujeto crítico con capacidad de producir conocimiento para transformar sus interrelaciones personales, comunitarias y su proyecto de vida” (Gómez‐Ramírez et al. 2023, p‐1) [“This article highlights among the results the experience of a young woman from the community, a university student, and the emergence of her habitus as a community researcher and critical subject with the capacity to produce knowledge to transform her personal and community interrelationships and her life project” (Gómez‐Ramírez et al. 2023, p‐1)]
			“la comprensión de la realidad de los jóvenes no debe partir solo de la mirada del experto académico, sino que el conocimiento y las reflexiones deben fundarse también en las comprensiones y significado de los jóvenes, apostándole a una construcción colectiva de la realidad” (López‐Taborda 2019, p. 8) [(Gómez‐Ramírez et al. 2023, p. 1)
			“Understanding the reality of young people should not only stem from the perspective of the academic expert, but knowledge and reflections should also be based on the understandings and meanings of young people, committing to a collective construction of reality.” (López‐Taborda 2019, p. 8)]

As can be seen in Table 2, in North America, three interrelated conceptions predominate regarding young people and their role in research processes. First, the recognition of their individual voice—distinct from the adult perspective—and their capacity for agency, which is achieved through the development of their critical awareness (Anderson et al. 2024; Levy et al. 2023; Rubin et al. 2017) and leads to individual empowerment, understood as “greater control over access to the conditions and resources that affect their lives” (Kohfeldt et al. 2011, p. 29). Second, they are seen as subjects who have the power to generate knowledge and can therefore participate as co‐researchers (Brown 2010; Davis 2016; Anderson 2020), challenging the arbitrary nature of power that restricts the role of researcher to the adult world (Cammarota and Fine 2008; Cammarota and Romero 2009; Kirshner and Pozzoboni 2011). Likewise, youth participation is conceived as focusing on their potential to contribute to the improvement of institutional systems, such as schools and public health programs (Anyon et al. 2018), but this process occurs in formal institutional contexts that operate under a hierarchical model of power distribution (Jacquez et al. 2013; Ozer et al. 2020), which sometimes leads to “limited empowerment” (Ozer et al. 2013, p. 75) and restricts transformative potential to incremental reforms (Levy et al. 2023; Stovall and Delgado 2009).

In Latin America, three perspectives also stand out, but with different orientations than those identified in North America. First, although, as in North American studies, young people are conceived as co‐creators of knowledge, this knowledge has two different characteristics: it is knowledge situated in their own realities and serves the community purposes of historical memory, resistance to oppression, and their struggles for the vindication of collective rights and structural transformation (Gómez‐Ramírez et al. 2023; González‐Quiroz and Pérez‐Cadavid 2018; Restrepo Galvis et al. 2023; Rodríguez‐Gaitán 2020). Emphasis is also placed on the contextual, social, and historical identity of young people, marked by armed conflict and the tension between stigmatization as violent and unproductive individuals (Vanegas 2018) and their emergence as collective and sociopolitical agents (Ariza‐Arias 2018; González‐Quiroz and Pérez‐Cadavid 2018). These research approaches challenge traditional hierarchies, emphasize horizontal collaboration between young people and adults (Frutuoso et al. 2022; Hincapié and Herrera 2014; Restrepo Galvis et al. 2023), and advocate for youth participation aimed at broad social change.

This divergence in conceptualization is also reflected in the operationalization of youth roles. In North America, youth tend to seek visibility and influence through structured, policy‐aligned engagements (Wilson et al. 2007; Rubin et al. 2017). In Latin America, youth contributions emphasize cultural regeneration, political resistance, and collective healing, rejecting top‐down models of participation in favor of community‐rooted praxis (Rodríguez‐Gaitán 2020; Pérez‐Velásquez 2019). These differences in how participation is operationalized point to deeper distinctions in the theoretical foundations and political contexts that shape participatory research across regions.

### 3.2 Theoretical and Political Orientation

This section addresses the research question concerning the similarities and differences between YPAR in North America and participatory research with young people in Latin America with respect to their theoretical and policy orientations. The findings reveal more pronounced regional differences than similarities. These differences are rooted in distinct theoretical traditions and political contexts, which, in turn, shape divergent approaches to YPAR and participatory research with young people.

In North America, YPAR is grounded in three primary theoretical orientations: (a) CRT and LatCrit, (b) Freirean critical pedagogy, and (c) theories of Positive Youth Development (PYD), often coupled with a pragmatic orientation toward institutional reform (Table 3). Within North American studies, Freire's notion of praxis, understood as the integration of critical reflection and action, is a recurring feature and is used to foreground the interpretation of social realities through participants' lived experiences. However, despite the frequent incorporation of critical theoretical perspectives, their application is often circumscribed by developmental frameworks, such as PYD, or by reformist efforts that seek change within existing institutional structures rather than more expansive structural transformation.

**Table 3:** Theoretical orientation in YPAR projects in North America and participatory research with youth in Latin America.

Theoretical approaches	Representative studies	Illustrative quotes
**North America**		
Critical Race Theory (CRT) and LatCrit	Akom et al. (2008), Call‐Cummings and Martinez (2016a), Cammarota and Romero (2009), Davis (2016), Fine (2009), Raygoza (2016)	“we use Critical Race Theory (CRT) as both an epistemological and methodological tool in evaluating racism in education in terms of those young people included in the dominant paradigm and those who are not” (Akom et al. 2008, p. 3)
Freirean Critical Pedagogy	Cammarota and Fine (2008), Davis (2016), Grace and Langhout (2014), King (2013), Kohfeldt et al. (2011), Malorni et al. (2022)	“Therefore, educational projects using critical pedagogy should not ‘deposit’ information about educational inequities into Black girls, as this approach does not allow students to engage in the fact‐finding and inquiry processes through dialogue. As Freire (1970) states, ‘only dialogue, which requires critical thinking, is also capable of generating critical thinking’ (p. 93)….” (Davis 2016, p. 24)
Positive Youth Development (PYD) and Institutional Reform	Anyon et al. (2018), Jacquez et al. (2013), Ozer et al. (2013), Rubin et al. (2017), Stovall and Delgado (2009)	“One avenue that might be promising for investigators interested in youth engagement outcomes is the Positive Youth Development (PYD) perspective. Like CBPR, PYD is a strength‐based approach that maintains the assumption that youth are not ‘problems to be fixed but resources to be developed’ (Lerner et al. 2005).”(Jacquez et al. 2013, p. 186)
**South America**		
Popular Education and Freirean Critical Pedagogy	Brandão Neto (2018), Gómez‐Villota et al. (2015), Gómez‐Ramírez et al. (2023), López‐Taborda (2019), Téllez‐Ballén (2019), Urrea‐Pérez (2014)	“O Método Paulo Freire: Círculos de Cultura tem como proposta fundamental desenvolver uma ação educativa como ato de recriação, de ressignificação dos aspectos da realidade. Este Método tem como fio condutor a reflexão‐ação visando à libertação, não somente no campo cognitivo, mas essencialmente nos campos social e político” (Brandão Neto 2018, p. 44) [“The Paulo Freire Method: Culture Circles has as its fundamental proposal to develop educational action as an act of recreation, of resignification of aspects of reality. This method has as its guiding thread reflection‐action aimed at liberation, not only in the cognitive field, but essentially in the social and political fields” (Brandão Neto 2018, p. 44)]
Decolonial theories, liberation psychology, and Southern thought	Ariza‐Arias (2018), González‐Morales (2019), Pérez‐Velásquez (2019), Restrepo Galvis et al. (2023), Rodríguez‐Gaitán (2020)	“El cuestionamiento al modelo de desarrollo capitalista extractivo… emerge de la necesidad de contribuir en superar y combatir las desigualdades que enfrentan sujetos étnicos y populares históricamente excluidos y marginalizados… relacionadas con las formas de vida de los pueblos étnicos, y esto se asocia a la lógica colonial que sustenta la desigualdad epistémica” (Rodríguez‐Gaitán 2020, p. 27) [“The questioning of the extractive capitalist development model… emerges from the need to contribute to overcoming and combating the inequalities faced by historically excluded and marginalized ethnic and popular subjects… related to the ways of life of ethnic peoples, and this is associated with the colonial logic that sustains epistemic inequality” (Rodríguez‐Gaitán 2020, p. 27)]
Latin American Critical Theory, Historical Materialism, and Participatory Action Research (PAR) principles	González‐Quiroz and Pérez‐Cadavid (2018), Hincapié and Herrera (2014), Lince‐Bohórquez (2010), Niquepa (2023), Otálvaro Castro et al. (2022), Vanegas (2018)	“corriente metodológica que se inspiraba en interpretaciones críticas y marxistas de la realidad. De un lado, esta nueva perspectiva propuso que el conocimiento y la acción podrían ser componentes de la transformación más potente si se producían de forma participativa y democrática (Ortiz and Borjas 2008). De otro lado, que era necesario situar este conocimiento al servicio de la emancipación y la liberación de los grupos sociales que sufrían condiciones de vida indignas por cuenta de las diferencias de clases.”(Gómez‐Ramírez et al. 2023, p. 9) [“A methodological current inspired by critical and Marxist interpretations of reality. On the one hand, this new perspective proposed that knowledge and action could be components of the most powerful transformation if they were produced in a participatory and democratic way (Ortiz and Borjas 2008). On the other hand, it was necessary to place this knowledge at the service of the emancipation and liberation of social groups that suffered undignified living conditions due to class differences.” (Gómez‐Ramírez et al. 2023, p. 9)]
Historical and cultural memory approaches	Iuri et al. (2019), Luján‐Cortés (2020), Naranjo‐Rojo et al. (2022)	“La recuperación y autoconocimiento crítico de la historia posibilita descubrir, a través de la memoria colectiva, elementos del pasado y del presente que han sido útiles en la lucha de los pueblos y que pueden ser usados en el presente y futuro. La tradición oral ocupa un lugar central aquí, y el papel de los mayores toma particular importancia y relevancia.” (Rodríguez‐Gaitán 2020, p. 175) [“The recovery and critical self‐knowledge of history makes it possible to discover, through collective memory, elements of the past and present that have been useful in the struggle of peoples and that can be used in the present and future. Oral tradition occupies a central place here, and the role of elders takes on particular importance and relevance.” (Rodríguez‐Gaitán 2020, p. 175)]

In terms of political foundations, studies in North America demonstrate a strong connection between theory and practice and invoke strong critical roots (CRT/LatCrit). However, the predominant political approach is geared toward influencing existing policies and institutions (schools, juvenile courts, public health agencies). Likewise, the empowerment perspective emphasizes individual agency, civic engagement, and leadership, which restricts the collective perspective of empowerment, which is often limited by institutional tensions. In this sense, Ozer et al. (2013) coin the term “bounded empowerment” to refer to “…experience of students of meaningful appropriation and control, in the context of significant power constraints.” (p. 19)

In Latin America, participatory research with young people coincides with several North American studies in its foundation in Popular Education and Freirean Critical Pedagogy. However, other theoretical frameworks are distinctively integrated, including decolonial theories, liberation psychology, and Southern thought; Latin American critical theory, historical materialism, and principles of PAR; as well as historical and cultural memory approaches. In addition to having a strong foundation in popular education, studies in Latin America integrate decolonial and global south frameworks, an approach oriented toward structural transformation and class/power analysis, and the use of memory and culture as research tools.

The adoption of theoretical trends in Latin America gives studies a clear political orientation towards critical awareness, radical social transformation, and cultural resistance. Latin American studies conceive of research as a political, pedagogical, critical, holistic, and alternative process that should serve the emancipation of exploited groups.

### 3.3 Methodological Structures and Youth Involvement

Regarding the third research question, which asked about the methodological similarities and differences between YPAR in North America and participatory research with youth in Latin America, particularly in relation to youth participation in the research process, the findings reveal substantial variation across regions. While the studies reviewed show a wide range of methodological differences, they also share several common methodological features related to participatory engagement.

In terms of shared characteristics, it can be said that studies in both regions develop methodological designs that aim to enable high youth participation in research processes (although there are differences in the conception of what such participation entails) and use techniques that seek to listen to youth voices. They also share methodological designs that advocate flexibility and use a wide mix of techniques for collecting and analyzing information. In this regard, the widespread use in both regions of visual methods, such as photographs, as a means of youth expression and reflection is noteworthy (Brandão Neto 2018; Costa et al. 2013; Davis 2016; Foster‐Fishman et al. 2010; Naranjo‐Rojo et al. 2022; Wilson et al. 2007).

With regard to differences, YPAR studies in North America exhibit more structured designs than those in Latin America, where there is a predominant concern for the use of standardized tools such as surveys, interviews, or formal qualitative methods (Fisette and Walton 2011; Goodnough 2014; Kirshner et al. 2011). For example, studies conducted in California, Michigan, and Texas emphasized youth‐facilitated surveys, photovoice, and public health assessments with adult facilitators, who provided research training and access to institutions (Akom et al. 2016; Anderson 2020; Foster‐Fishman et al. 2005, 2010; Jacquez et al. 2013). In California, youth also used Photovoice to document school violence and engage policymakers through visual storytelling (Wilson et al. 2007). With training and guidance from their teachers and a university research team, students participated in a structured research phase in which they examined the issue using multiple methods. As described by Ozer et al. (2013), “with training and guidance from their teachers and the university team, students engaged in a research phase to study and understand the problem, using a range of survey, interview, observational, and multi‐media approaches for data collection and analysis as determined by each group of student researchers” (p. 16). Photovoice, in turn, allowed participants to visually represent their lived experiences, as “participants represent their world with their own photographs, which they then analyze to surface their meaning” (Wilson et al. 2007, p. 242).

To this end, YPAR studies often develop institutionalized training processes that prepare young people to administer research instruments with their peers and analyze the resulting data (Davis 2016; King 2013; Raygoza 2016). In many cases, research is formally embedded within the school curriculum and recognized as an academic course or credit requirement (Cammarota 2007a; Cammarota and Romero 2009). As Cammarota (2007a) explains, “the curriculum provides students with all their social sciences credits for graduation. They meet everyday for one period and for four consecutive semesters… [to] conduct participatory action research” (p. 343).

In contrast, studies in Latin America used fewer schematic designs that made it possible to place ancestral/peasant knowledge and academic knowledge on the same level. Participatory research with young people in Latin America favors the use of techniques focused on the territory and based on the community and on the recovery of experiences, indigenous knowledge, and collective memory. These approaches prioritize research, integrating it into local epistemologies and activist traditions (Ariza‐Arias 2018; González‐Morales 2019; Pérez Velásquez 2019; Restrepo Galvis et al. 2023; Rodríguez‐Gaitán 2020). In Colombia and Brazil, youth‐led initiatives used storytelling and the arts to recover displaced narratives, resist territorial injustice, and support cultural continuity (Frutuoso et al. 2022; Pérez Velásquez 2019; Restrepo Galvis et al. 2023; Rodríguez‐Gaitán 2020).

Intensive use is made of different artistic expressions such as theater, dance, oral narratives, and painting as tools for research and not only for expression (Brandão Neto et al. 2020; Lujan‐Cortés 2020; Niquepa 2023; Vanderbilt Hernández 2020). Some research even claimed that knowledge is produced through the experiences, emotions, and movements that participants experience in their skin, that is, research is conducted “from the body” (Esguerra‐Moreno and Roa‐Velandia 2015; Naranjo‐Rojo et al. 2022; Restrepo Galvis et al. 2023). In Brazil, youth used counter‐mapping and participatory theater to resist environmental injustice and advocate for indigenous land claims (Brandão Neto et al. 2020; Frutuoso et al. 2022). Colombian youth documented armed conflict and displacement, constructing community archives to support collective healing and advocacy (Restrepo Galvis et al. 2023; Rodríguez‐Gaitán 2020; Vanegas 2018).

These epistemological and methodological orientations are further articulated by authors through their descriptions of how knowledge is generated and embodied within participatory research processes. The following excerpts illustrate the emphasis on place‐based, embodied, and relational forms of knowing that underpin these approaches.

“[se utilizaron] técnicas que permitieron la conversación, la lectura, la escucha y el recorrido, facilitando develar asuntos epistémicos… donde se entiende la tierra como un ser vivo” (Pérez‐Velásquez 2019, p. 11) [“(Techniques were used) that allowed for conversation, reading, listening, and exploration, facilitating the unveiling of epistemic issues… where the earth is understood as a living being” (Pérez‐Velásquez 2019, p. 11)]

“[la investigación] promueve la revalorización de los saberes tradicionales, ancestrales y locales… El conocimiento creado a partir de esta metodología es por medio de la reflexión y presencia en la vida diaria o cotidiana de las comunidades” (González‐Morales 2019, p. 106) [“(The research) promotes the revaluation of traditional, ancestral and local knowledge… The knowledge created from this methodology is through reflection and presence in the daily or everyday life of the communities” (González‐Morales 2019, p. 106)]

“La subjetividad habita en el cuerpo, las corpografías pueden ser entendidas como el análisis y reflexión, disertación, investigación y creación a partir de los lenguajes corporales… en este sentido nos referimos a interpretar las diferentes voces, resonancias, vibraciones, visceralidades, organicidades, desde los biológico, lo anatómico, lo biomédico, pero también, desde las ciencias sociales, para poner allí preguntas sobre lo político y cultural…” (Naranjo‐Rojo et al. 2022, p. 23) [“Subjectivity resides in the body; corpographies can be understood as the analysis and reflection, discourse, research, and creation based on body languages… in this sense, we refer to interpreting the different voices, resonances, vibrations, visceral aspects, and organicities, from the biological, the anatomical, the biomedical, but also from the social sciences, in order to raise questions about the political and cultural…”(Naranjo‐Rojo et al. 2022, p. 23)]

Using Arnstein's Ladder of Citizen Participation (1969), youth participation in North American YPAR studies was classified for us into four distinct categories: *Complete Protagonism*, *Partial Protagonism*, *Reflexive Participants*, and *Informants*. In NA, the average participation score was 4.35 (SD = 1.12), with 34 projects achieving a score of 5.0—signifying full protagonism, including research leadership and advocacy. Additional studies showed partial protagonism (*n* = 9), reflexive participation (*n* = 8), and informant roles (*n* = 5) (Table 4). In Latin América, the assessment of youth roles were more evenly distributed across lower tiers of Arnstein's Ladder of Citizen Participation: our analysis categorized 13 studies as employing youth primarily as informants, 6 as reflexive participants, 6 as demonstrating partial protagonism, and only 4 studies reached full protagonism (Table 4).

**Table 4:** Classification of YPAR studies in North America and Participatory Research with Youth in Latin America according to the level of participation in accordance with Arnstein's ladder of participation (1969).

Participation level assessment	Region	Studies
Complete Protagonism	NA	**34 studies** (Akom et al. 2016, 2008, 2016, 2008; Anderson 2020; Anderson et al. 2024; Anyon et al. 2018; Branquinho et al. 2020; Brown 2010; Call‐Cummings and Martinez 2016a; Cammarota 2007b, 2017, 2007b, 2017; Cammarota and Fine 2008; Cammarota and Romero 2011, 2009, 2011, 2009; Conrad 2015; Cox 2017; Davis 2016; Desai 2019; Fine 2009; Fisette and Walton 2011; Foster‐Fishman et al. 2010; Goessling and Doyle 2009; Grace and Langhout 2014; Jacquez et al. 2013; King 2013; Kirshner et al. 2011; Kohfeldt et al. 2011; Levy et al. 2023; LPC Consulting Associates, Inc 2015; Maker Castro et al. 2022; Malorni et al. 2022; Nakanishi 2009; Ozer et al. 2020; Valdez et al. 2019; Wanis 2010)
	LA	**4 studies** (Gómez‐Villota et al. 2015; González‐Quiroz and Pérez‐Cadavid 2018; Luján‐Cortés 2020; Rodríguez‐Gaitán 2020)
Partial Protagonism	NA	**9 studies** (Call‐Cummings and Martinez 2016b; Goodnough 2014; Kirshner and Pozzoboni 2011; Raygoza 2016; Rubin et al. 2017; S. J. Tanner 2017; Vaccarino‐Ruiz et al. 2022; Wilson et al. 2008; Zenkov et al. 2013)
	LA	**6 studies** (Brandão Neto 2018; Brandão Neto et al. 2020; Frutuoso et al. 2022; Niquepa 2023; Otálvaro Castro et al. 2022; Téllez‐Ballén 2019)
Reflexive Participants	NA	**8 studies** (Foster‐Fishman et al. 2005; Ginwright 2007; Haskie‐Mendoza et al. 2018; Ozer et al. 2013; Ozer and Douglas 2013; Wang and Pies 2004; Wilson et al. 2007; Yang 2009)
	LA	**6 studies** (Costa et al. 2013; Esguerra‐Moreno and Roa‐Velandia 2015; Hincapié and Herrera 2014; JiménezVallejo and ValadezFigueroa 2003; Lince‐Bohórquez 2010; López‐Taborda 2019)
Informants	NA	**5 studies** (Cammarota 2007a; deLara 2002; Desai and Abeita 2017; Stovall and Delgado 2009; S. Tanner and Corrie 2016)
	LA	**13 studies** (Ariza‐Arias 2018; Barrantes‐Rojas 2012; Gómez‐Ramírez et al. 2023; González‐Morales 2019; Iuri et al. 2019; Naranjo‐Rojo et al. 2022; Novoa and y Poveda Garrido 2020; Ospina‐Álvarez 2022; Pérez‐Velásquez 2019; Restrepo Galvis et al. 2023; Urrea‐Pérez 2014; Vanderbilt Hernández 2020; Vanegas 2018)

### 3.4 Purpose and Praxis of YPAR

Finally, in response to the last question, “Do YPAR in North America and participatory research with young people in Latin America differ in terms of their objectives and practices?,” the results indicate that despite methodological and ethical similarities in both regions, there are also clear regional differences in the “why?” of the research.

In terms of similarities, studies conducted in both North America and Latin America recognize that young people possess unique and situated knowledge about their lived realities (Akom et al. 2016; Brandão Neto et al. 2020). This recognition underpins a shared commitment to challenging adult‐centrism and disrupting traditional adult–youth hierarchies in favor of more horizontal and collaborative forms of interaction (Cox 2017; Gómez‐Ramírez et al. 2023; Goodnough 2014; Kohfeldt et al. 2011). A further point of convergence lies in the emphasis on research that goes beyond data collection and knowledge generation to actively contribute to social change. However, important differences emerge in the scope and orientation of this change. In North American studies, change is most often conceptualized at the institutional level, such as through modifications to school rules or policies (Ozer et al. 2013), whereas in Latin American studies, social change is more frequently framed in terms of structural transformation, including shifts in consciousness and the defense of territory (Rodríguez‐Gaitán 2020).

These points of convergence and divergence are reflected in how scholars conceptualize the role and purpose of YPAR and PAR across contexts. Authors explicitly describe research as a political and transformative practice that centers youth knowledge and seeks to move beyond extractive approaches. The excerpts below foreground how these commitments are articulated in the literature, while also revealing variation in how social change is envisioned, from institutionally focused reforms to broader efforts aimed at transforming consciousness, power relations, and territorial realities.

“The intention is for students to reclaim the political space that silences their voices by filling in the missing element, student knowledge, for developing effective policies for young people” (Cammarota and Romero 2009, p. 54)

“the purpose of YPAR is to actively intervene in order to change knowledge and practices to improve the lives of youth and their communities” (Anyon et al. 2018, p. 865)

“No solo se trata de situar el contexto como un problema de investigación, sino de incidir en este, de tal modo que el conocimiento que se produce permita construir puentes hacia la acción” (Rodríguez‐Gaitán 2020, p. 8) [“It is not only about situating the context as a research problem, but about influencing it, in such a way that the knowledge produced allows building bridges towards action” (Rodríguez‐Gaitán 2020, p. 8)]

“[la IAP] no se encuentra orientada exclusivamente al fetiche de la investigación, no es investigar por investigar como un fin en sí mismo […] Las metas del conocimiento liberador y del poder político de la IAP son dos… adquirir suficiente creatividad y fuerza transformadora, las que se deben expresar en proyectos regionales, en acciones, en políticas y en luchas de la resistencia.” (González‐Quiroz and Pérez‐Cadavid 2018, p. 44) [“[PAR] is not exclusively oriented towards the fetish of research, it is not research for research's sake as an end in itself […] The goals of liberating knowledge and the political power of PAR are twofold… to acquire sufficient creativity and transformative force, which must be expressed in regional projects, in actions, in policies and in resistance struggles.” (González‐Quiroz and Pérez‐Cadavid 2018, p. 44)]

Thirdly, studies in both regions use art for data collection and action strategies, although this use is broader and more diverse in Latin American studies, where theater, muralism, social cartography, dance, drawing, among others, are used (Esguerra‐Moreno and Roa‐Velandia 2015; Lujan‐Cortés 2020; Vanderbilt Hernández 2020), while in North American studies, Photovoice predominates almost exclusively (Foster‐Fishman et al. 2010; Wilson et al. 2007).

However, the major difference between North American and Latin American studies lies in their purposes. In general, studies in North America aim to improve or reform institutions and social policies by generating evidence and producing reports or presentations. Such is the case with studies that sought to evaluate and adjust school policies, curriculum, and school climate (Cammarota and Romero 2011; Kirshner and Pozzoboni 2011; Ozer et al. 2013); or intervening in specific health risk factors such as obesity, substance use, and violence by changing social norms or the physical environment (Valdez et al. 2019; Wilson et al. 2007); or promoting the social inclusion of excluded youth (Brown 2010; King 2013). Another common purpose in North American studies was the promotion of positive youth development by fostering individual skills such as leadership, self‐efficacy, civic competence, and academic/college readiness (Jacquez et al. 2013; Ozer and Douglas 2013).

These institutional and developmental orientations are further articulated in how scholars describe the intended outcomes of YPAR in North American contexts. The excerpts below emphasize engagement with schools and communities as sites for improvement, alongside an explicit focus on individual‐level developmental benefits for participating youth.

“YPAR is becoming increasingly common as a means of promoting urban young people's engagement in improving their schools and communities… In addition to improving community settings and resources—such as schools, neighborhoods, and agencies that serve youth—the YPAR process is intended to yield developmental benefits for the young people who participate” (Ozer et al. 2013, pp. 13‐14)

“For traditionally marginalized youth who are often positioned as ‘underachieving,’ YPAR is a particularly powerful avenue for personal growth and societal change, providing them with an alternative to the ‘constrictive patterns of schooling’… and positioning them as motivated, engaged, and successful members of their schools or communities” (King 2013, p. 17)

In contrast, studies in Latin America affirm social and political transformation as their purpose. Following the tradition of Popular Education, the central purpose of several studies was the transformation of structural conditions of inequality and the “liberation” or “emancipation” of oppressed subjects (Brandão Neto 2018; Gómez‐Ramírez et al. 2023; González‐Quiroz and Pérez‐Cadavid 2018), or to recover cultural narratives and support sovereignty over the territory and its natural resources through the recovery of memory and the strengthening of territorial identity (Ariza‐Arias 2018; Brandão Neto et al. 2020; González‐Morales 2019; Pérez‐Velásquez 2019, 2019; Rodríguez‐Gaitán 2020). Other studies are also set in contexts of armed conflict and systemic violence or in urban settings, whose purposes revolved around shaping political subjectivities in young people by documenting violence and displacement and promoting individual and collective awareness (Esguerra‐Moreno and Roa‐Velandia 2015; Gómez‐Ramírez et al. 2023; Vanegas 2018), but also aspired to rebuild the social fabric, heal collective traumas, and generate a culture of peace from the community level (Naranjo‐Rojo et al. 2022; Téllez‐Ballén 2019). These initiatives often involve young people as part of collective resistance movements, in which research is inseparable from the struggle and liberation of the community.

These transformative and resistance‐oriented purposes are further illustrated in how authors describe the goals of their projects. The excerpts below emphasize knowledge production as a collective, situated, and explicitly political practice aimed at territorial defense, the formation of political subjectivities, and community transformation.

“El objetivo general de este proceso formativo es ‘promover que docentes, jóvenes, líderes y lideresas se conviertan en agentes del conocimiento intercultural, con capacidad de producir conocimientos contextualizados sobre la situación de los grupos étnicos en sus territorios y actuar articuladamente para la defensa territorial’” (Rodríguez‐Gaitán 2020, p. 43) [“The overall objective of this training process is to ‘promote that teachers, young people, leaders and female leaders become agents of intercultural knowledge, with the capacity to produce contextualized knowledge about the situation of ethnic groups in their territories and to act in an articulated manner for territorial defense’” (Rodríguez‐Gaitán 2020, p. 43)]

“La presente investigación plantea una postura social, en la medida que pretende visibilizar a los jóvenes, no desde las problemáticas sociales que los atañen, sino desde la constitución de sus subjetividades políticas, que a la vez generan acciones de participación colectiva, que les permiten configurarse como actores sociales que aportan desde una postura crítica ideas de cambio y transformación social, en aras de generar aportes y beneficio a sus comunidades” (Vanegas 2018, p. 10) [“The overall objective of this training process is to ‘promote that teachers, young people, leaders and female leaders become agents of intercultural knowledge, with the capacity to produce contextualized knowledge about the situation of ethnic groups in their territories and to act in an articulated manner for territorial defense’” (Rodríguez‐Gaitán 2020, p. 43)]

## 4 Discussion

The purpose of this study was to compare YPAR across North America and Latin America (known as PAR with young people in Latin America). The results indicate significant theoretical, methodological, and political differences between the two regions, as well as a range of youth involvement in the research process. This discussion will explore the implications of these findings for theory, practice, and future research, as well as identify the lessons learned for improving the practice of participatory action research with young people across regions.

### 4.1 Theoretical and Conceptual Convergences and Divergences in Participatory Research With Young People

The findings demonstrate a strong convergence in the application of Paulo Freire's theoretical contributions within YPAR studies conducted in both North America and Latin America. Across regions, Freire's work emerges as the most consistent epistemological, methodological, and theoretical foundation underpinning participatory research with young people. In this sense, Freire's critical pedagogy functions as a central bridge linking YPAR traditions in North America and Latin America, providing a shared framework for how knowledge is produced, how relationships between youth and adults are structured, and how research is oriented toward social transformation (Freire 1980; Giroux 2001).

First, studies across both regions consistently invoke Freire's concept of praxis, understood as the inseparable relationship between knowledge construction and transformative action. Rather than treating research as an end, YPAR projects emphasize the production of knowledge that is directly tied to collective action and social change, reinforcing research as an inherently political and interventionist practice (Brandão Neto 2018; Cammarota and Fine 2008; Gómez‐Ramírez et al. 2023; Levy et al. 2023).

Second, the critique of vertical or “banking” education is foundational to YPAR in both North American and Latin American contexts. Traditional models in which adults unilaterally transmit knowledge to passive youth are explicitly rejected in favor of participatory and horizontal approaches that redistribute epistemic authority. These methodologies challenge adult‐centered hierarchies by positioning young people as co‐researchers whose lived experiences are central to the research process (Cammarota and Romero 2009; Esguerra‐Moreno and Roa‐Velandia 2015; Grace and Langhout 2014; Urrea‐Pérez 2014).

Third, horizontal dialogue is consistently emphasized as a core methodological strategy for fostering participation and validating youth knowledge. Dialogic practices are used to disrupt silence, surface experiential knowledge, and support collective meaning‐making, allowing young people to engage critically with their realities and with one another as equal contributors to research processes (Brandão Neto 2018; Malorni et al. 2022; Téllez‐Ballén 2019).

Relatedly, the promotion of critical awareness (i.e. conscientização) represents a shared objective across YPAR studies in North America and Latin America. Research processes are intentionally designed to support young people in moving beyond passive acceptance of social conditions toward a critical understanding of the structural forms of oppression that shape their lives. This heightened critical awareness is positioned as a precursor to action, framing investigative praxis as a mechanism through which young people collectively work to transform unjust conditions (Davis 2016; Malorni et al. 2022; Rodríguez‐Gaitán 2020; Téllez‐Ballén 2019).

Despite sharing roots in Freirean thought, one of the most notable findings is the difference between North American and Latin American studies in terms of their purposes and the theoretical frameworks used to conceptualize research and youth. In North America, young people were conceptualized primarily as agents of individual empowerment within educational and institutional settings, hence studies in this region focused on improving institutional systems, such as schools and public health programs (Anyon et al. 2018; Kirshner and Pozzoboni 2011; Ozer et al. 2020). This approach aligns with a reformist policy orientation, in which young people work within established frameworks to promote change (Ozer et al. 2013; Rubin et al. 2017).

In contrast, Latin American studies fundamentally view young people as political agents and co‐creators of knowledge, hence the emphasis in Latin American studies is not on change within institutional systems, but rather on community‐driven activism aimed at social transformation (González‐Quiroz and Pérez‐Cadavid 2018). Projects thus focused on addressing structural inequalities through activist movements, often related to land rights, indigenous rights, and historical memory (Ariza‐Arias 2018; Pérez‐Velásquez 2019; Rodríguez‐Gaitán 2020). The political orientation in Latin America reflects a radical stance, emphasizing social justice and community empowerment rather than individual or institutional change.

These findings suggest that YPAR in North America tends to align more with individual empowerment and seeks policy‐oriented reforms within institutional systems, while Latin America adopts a more collective, community‐oriented approach focused on social transformation and activism (Díaz‐Arévalo 2022). These distinctions reflect the regional histories of settler colonialism in North America and postcolonial resistance in Latin America, which shape how youth participation is theorized and practiced (Delgado and Staples 2007; Domínguez and Cammarota 2022; Lee et al. 2023). These differences highlight the need to further explore how regional contexts, including political, cultural, and historical factors, shape how youth are conceptualized in YPAR initiatives.

### 4.2 Youth Participation: Levels and Roles

The findings reveal clear distinctions in the conceptualization and practice of YPAR. In North America, although YPAR is influenced by critical pedagogy (McLaren and Kincheloe 2007) and critical race theory (Solórzano and Yosso 2002), in which young people are considered critical co‐researchers and advocates for social justice, its purpose is often linked to individual empowerment and singular, institutionalized changes. This vision is connected to psychological theories of empowerment (such as intrapersonal agency) and the advocacy of youth voices (Anyon et al. 2018; Jacquez et al. 2013; Kirshner and Pozzoboni 2011; Ozer et al. 2013). In contrast, projects in Latin America take a more collective and community‐based approach, where youth leadership is framed as a shared political process of resistance and radical social transformation, deeply rooted in the traditions of popular education (González‐Quiroz and Pérez‐Cadavid 2018; Rodríguez‐Gaitán 2020; Urrea‐Pérez 2014; Vanegas 2018).

The results regarding youth participation reveal a marked contrast between the two regions, not only in terms of theoretical orientation, but also in terms of levels of involvement in the research process. In North America, a significant proportion of studies classified young people as protagonists, as they were fully involved in research design, data collection, and action. These studies reflect a strong emphasis on youth leadership within established institutional frameworks, in which young people are seen as active agents of reform.

In Latin America, however, young people were more commonly classified as “consulted” or “involved,” and their roles often focused on data collection and community‐based research. Only a few studies (4 out of 29) classified young people as protagonists, where they took on leadership roles in community projects aimed at addressing social justice issues. This reflects a more collective approach to youth participation, in which young people are positioned as part of a broader community effort for social change, rather than as individual leaders. These differences suggest that while North American YPAR tends to emphasize individual empowerment and leadership among young people, Latin American YPAR places greater emphasis on collective action and community mobilization, in which young people are involved.

The broader structural context of youth rights may also help explain these patterns. Latin American countries, which have widely ratified the United Nations Convention on the Rights of the Child, often incorporate youth participation mandates into education and social policy frameworks, which may facilitate collective and rights‐based approaches to YPAR. In contrast, the United States is the only country in the Americas, and the only UN member state, that has signed but not ratified the Convention on the Rights of the Child (United Nations Treaty Collection n.d.). This lack of formal commitment may reflect and reinforce the more individualistic and reform‐oriented forms of youth participation observed in the North American sample (Checkoway 2011; Larson 2000). These distinctions suggest that legal and institutional commitments to youth rights can significantly influence models and expectations of participatory research across regions.

The Ladder of Citizen Participation (Arnstein 1969) proved to be a useful tool for assessing these levels of participation, as it allows us to see the differences in orientation, institutional versus community‐based, of participatory research efforts with young people in both regions. Future studies could further explore the dynamics of youth participation by incorporating a deeper understanding of the cultural and political factors that influence how young people engage in community‐based research processes.

### 4.3 The Role of Youth in Knowledge Production

The findings underscore the critical role of youth as co‐creators of knowledge in YPAR. In North America, youth were involved in data collection, analysis, and dissemination, with a focus on addressing local issues such as school climate, health disparities, and community safety (Akom et al. 2016; Cammarota and Fine 2008; Foster‐Fishman et al. 2010; Jacquez et al. 2013). In Latin America, however, youth played a similar role but within a much broader activist framework, where their research efforts were aimed at addressing structural inequalities related to land rights, historical memory, promoting political participation and cultural preservation (Ariza‐Arias 2018; González‐Quiroz and Pérez‐Cadavid 2018; López‐Taborda 2019; Naranjo‐Rojo et al. 2022; Niquepa 2023; Otálvaro Castro et al. 2022; Pérez Velásquez 2019; Restrepo Galvis et al. 2023; Rodríguez‐Gaitán 2020; Vanegas 2018). These differences reflect the diverse goals of YPAR in the two regions: North American studies often aim for institutional reform and youth empowerment, while Latin American studies focus on social justice, community‐based action, and political resistance.

This contrast in youth roles also points to the varying conceptualizations of youth participation across regions. In North America, youth are often viewed as empowered individuals capable of influencing systemic change, while in Latin America, youth are conceptualized as members of collective movements working together to address community‐based issues. This divergence highlights the importance of regional contexts in shaping how youth are integrated into the research process and how their contributions are valued.

### 4.4 Implications for Practice, Policy, and Research

The findings of this review offer critical insights to inform the future of PAR with young people across the Americas. By highlighting the regional distinctions in theoretical orientation, project goals, and youth roles, this study encourages the refinement of methodologies, promotes context‐sensitive adaptations, and supports meaningful transnational collaboration. These implications are intended to guide the evolution of YPAR practice, policy, and research in ways that reflect the lived realities and aspirations of young people, while also facilitating the exchange of best practices across hemispheric boundaries.

#### 4.4.1 Policy Implications

In North America, where YPAR is typically embedded within institutional systems such as schools, health agencies, and nonprofit organizations, policies should formalize youth participation by integrating it into standard decision‐making structures. Studies by Foster‐Fishman et al. (2005) and Jacquez et al. (2013) show how institutionalized support for youth‐led data collection and advocacy can drive tangible policy shifts. Governments and school districts should consider creating and funding permanent youth advisory bodies, participatory grantmaking programs, and school‐based research hubs to sustain youth involvement in shaping policy.

In Latin America, where YPAR is often rooted in popular education and resistance movements, policy efforts should recognize and protect youth‐led community‐based initiatives. Research by Brandão Neto et al. (2020) and Restrepo Galvis et al. (2023) demonstrates how youth use participatory methods to challenge land dispossession and reclaim cultural memory. Policies that protect youth organizers, ensure equitable access to public funding, and legitimize nonformal education and community research can significantly amplify the reach and impact of these efforts.

Importantly, cross‐regional policy platforms that support North‐South collaborations such as UNESCO's Global Citizenship Education framework (UNESCO 2015), could serve as mechanisms to align institutional and grassroots approaches. These frameworks can provide space for bi‐directional learning and recognition of the diverse ways youth produce knowledge and effect change.

#### 4.4.2 Practice Implications

Practitioners in both North and Latin America should adopt flexible, participatory strategies that align with their regional contexts while also embracing hybrid models that draw from each other's strengths. In North America, where YPAR is often constrained by institutional timelines and bureaucratic structures, integrating culturally responsive practices from Latin American contexts, such as dialogical pedagogy, counter‐mapping, and participatory theater, can deepen community engagement and challenge power asymmetries (Cammarota and Romero 2011; Rodríguez‐Gaitán 2020). For example, scholars such as Delgado and Staples (2008) have advocated for more relational and culturally grounded practices in U.S.‐based youth development, drawing inspiration from Latin American traditions of resistance and collective action.

Conversely, practitioners in Latin America could benefit from adopting capacity‐building tools commonly used in the North American context, such as structured training in research methods, leadership development workshops, and youth‐authored policy briefs (Ozer et al. 2020; Rubin et al. 2017). These strategies can strengthen the technical aspects of youth‐led research while preserving its community‐rooted ethos. Studies like those by Cahill (2007) show that blending technical rigor with critical pedagogy enhances both the credibility and impact of YPAR projects.

Collaborative projects that intentionally merge different educational approaches have demonstrated success. For example, the U.S. Department of State's Youth Exchange and Study (YES) program illustrates a cross‐border youth exchange where American and Mexican students participate in initiatives that blend U.S.‐style advocacy training with immersive community experiences common in Latin American educational practices. Such hybrid models offer a promising blueprint for fostering transnational solidarity and shared political education (U.S. Department of State, Bureau of Educational and Cultural Affairs n.d.).

#### 4.4.3 Research Implications

Future research should explore how these cross‐regional collaborations function in practice and what tensions or innovations emerge when differing YPAR paradigms are brought into dialogue. There is a pressing need for studies that examine the long‐term impacts of youth participation on both individual trajectories and community change, especially in transnational contexts.

Comparative studies should also examine how intersecting identities—such as race, gender, class, and immigration status—influence youth experiences of power, voice, and inclusion in participatory research. As authors like Tuck (2009) and Fine and Torre (2019) argue, attention to the relational and politicized nature of knowledge production is critical to avoiding extractive or superficial engagements with marginalized communities.

Finally, methodological innovation must remain a priority. Researchers should work collaboratively with youth to develop tools that reflect informal, arts‐based, and nonverbal ways of knowing—particularly in settings where traditional academic methods may not capture the full depth of youth experience. Efforts such as those described by Wilson et al. (2007) and Anyon et al. (2018) demonstrate the value of mixed‐methods and participatory evaluation tools for amplifying youth perspectives.

## 5 Conclusion

This study provides a comprehensive comparative analysis of YPAR in North America and Latin America, shedding light on the theoretical, methodological, and socio‐political differences between the two regions. While North America YPAR emphasizes individual empowerment within institutional settings, Latin America PAR with young people prioritizes collective action and community mobilization for social transformation. The findings highlight the importance of contextualizing youth participation within the specific socio‐political and cultural landscapes of each region, while also suggesting opportunities for cross‐regional learning and collaboration. Future research should build on these insights to refine participatory methodologies and ensure that youth are fully empowered to contribute to meaningful social change.
